# Reply on Behalf of the Class

**Published:** 1870-03

**Authors:** H. K. Lathrop


### REPLY ON BEHALF OF THE CLASS.

BY H. K. LATHROP.

*Gentlemen :*

The Twenty-fourth Annual Session of the Ohio Dental College has now drawn to a close, and by the proceedings of this evening we are reminded that the time has come for us to separate. Many strong ties of friendship have been formed between us, as teachers and pupils, these now must be severed, and it must be with sadness that we part with those with whom we have been so pleasantly associated for so long a time. We have looked forward to this occasion with great anxiety; but now that we have received our reward, it will be with the greatest pleasure that we look back to it. It will be with great pleasure that we shall look back upon the two winters spent in attending the course of instruction given in this Institution, for we feel that we have been more than paid for the time and money spent in attendance here, for we have gained a knowledge of the profession that could not otherwise have been gained in years.

Gentlemen of the Faculty, these diplomas that we have received this evening we prize very highly, and hope so to use them as to do honor to those who gave them, and credit to ourselves. They are the best credentials we can get, for they were earned by hard study, and given by those who are known and acknowledged to be fathers in the profession.

You have our heart-felt thanks for the honor you have done us in giving us these diplomas; we also thank you for the interest you have taken in our studies, for the unselfish interest in our advancement, and the progress of true Dental science.

We know that your reward is not a pecuniary one, and we shall always cherish the kindest recollections of you all for this sacrifice on your part. Let me assure you, gentlemen, that words fail to express our gratitude for the kind instructions that we have received at your hands.

This winter, which at first promised to be so long, and full of trials, you have made very short and most pleasant; you have so kept our interest up, that, day by day, and week by week, the time has sped away, and now, before we can scarcely realize it, we are called together for the last time. The parting is like leaving a parent's home—it is with a shudder that we step out into the world to battle for ourselves; we have been so used to asking your advice in our every step, that it is almost like being cast out at sea in a small boat, without a compass, which direction to take one scarcely knows, although when we were shown the way it seemed very plain and easy to follow; but when left alone, the way seems rather dark. But the progress of the past is our encouragement for the future—for what has been done by others we can do, provided we take hold of it with true courage, and a will to succeed. I trust that we will so cultivate the principles you have taught us, that we may join with, and assist, you in that which is to come.

We pledge ourselves to be worthy members of the Profession, and we hope to be able to aid you in elevating the standard of our profession. We know that you have been laboring, for the benefit of science and the profession, for years, and some of you have grown old long before you should, on account of your unceasing energy in bringing to light unknown facts; and we hope to conduct ourselves in a manner worthy of being called brothers in the cause; or rather we shall consider ourselves more as your sons in the profession.

Respected Professors, in behalf of the graduating class, I bid you farewell.

Fellow graduates, I scarcely know what to say to you. Five months ago we met, most of us, as strangers; but we have met so often, and have been so pleasantly associated, that we have become like a band of brothers. The ties that bind us together have been daily growing stronger, until now it is with great reluctance that I say the time has come for us to part, for our paths diverge so, that, in all probability, many of us will never meet again. But we have a medium through which we can watch each others' movements to a greater or less extent, and that is, through the columns of the DENTAL REGISTER, in which I hope your names will often appear.

Gentlemen, our kind instructors have this evening declared us no longer their pupils; but let us continue to be students if we are no longer pupils. Let this be but the commencement of our studies, as well as the commencement of our professional career. Let us study with more vigor than ever, for we have a great work to perform, and we have a vast field for labor before us, and the opposition that we will meet and have to overcome, will be great.

Let the class that leaves this institution to-night, make its mark in the world, and strive to have this world better for our having lived in it. Let us not be like thousands that are entering the profession, who are but a burden to it, like barnacles on the bottom of a ship, that do nothing but impede its progress, for there is already altogether too many of this class in the profession. But, as Daniel Webster told the law student, when asked if there was room in the profession, said that there was plenty of room in the upper story. I hope no one of us will be satisfied with anything short of the upper story. Then let our motto be, onward and upward!

We have advantages that our worthy instructors did not have when they commenced the practice of our profession, for we have the benefit of their life experience and experiments; besides, the instruments and appliances that they had then are not to be compared with what we have to-day; but, as Dentistry is not one of the most easy professions to follow, it will not only require us to be constantly willing, but will cause us many anxious days and sleepless nights.

There is no calling or profession that requires the constant use of all the faculties as Dentistry does. To keep up to the times, it will be necessary for us to patronize the literature of our profession, and read, as well as experiment, for ourselves, for there are new things coming up every day, which we will have to test for ourselves.

Dear classmates, lest I weary you by saying more, let me say, in conclusion, let us strive to be good men and good Dentists; to perform well the task that has been given us, and if we do not receive our reward in this world, we certainly will in the next.

Fellow students, let me say to you that it is but a short time until you will occupy our places—a year will soon glide by; and to you who are looking forward to a realization of your hopes, another commencement day, let me say, let it not be a year idly spent, for lost time can never be regained. We go in advance of you but a short distance, and we hope ere long to meet you as co-workers in the field for labor, and may success greet you in all your efforts.

Respected Professors, fellow graduates and students, one and all, I bid you farewell.

